# The mechanism of dendritic cell-T cell crosstalk in rheumatoid arthritis

**DOI:** 10.1186/s13075-023-03159-8

**Published:** 2023-10-05

**Authors:** Zhandong Wang, Jinlong Zhang, Fangyu An, Jie Zhang, Xiangrui Meng, Shiqing Liu, Ruoliu Xia, Gang Wang, Chunlu Yan

**Affiliations:** 1 School of Traditional Chinese and Western Medicine, Gansu University of Chinese Medicine, Lanzhou, Gansu 730000 China; 2 The First Clinical Medical College, Gansu University of Chinese Medicine, Lanzhou, Gansu 730000 China; 3 Teaching Experiment Training Center, Gansu University of Chinese Medicine, Lanzhou, Gansu 730000 China; 4 School of Basic Medicine, Gansu University of Chinese Medicine, Lanzhou, Gansu 730000 China; 5 Rheumatism and Orthopaedics Department, Affiliated Hospital of Gansu University of Chinese Medicine, Lanzhou, Gansu 730000 China

**Keywords:** Rheumatoid arthritis, Dendritic cells, Chemokines, PD-L1/PD-1 signalling axis, TGF-β signalling axis, Crosstalk mechanism

## Abstract

Rheumatoid arthritis (RA) is a chronic inflammatory disease characterised by joint pain and swelling, synovial hyperplasia, cartilage damage, and bone destruction. The mechanisms of dendritic cell (DC) and T cell-mediated crosstalk have gradually become a focus of attention. DCs regulate the proliferation and differentiation of CD4^+^ T cell subtypes through different cytokines, surface molecules, and antigen presentation. DC-T cell crosstalk also blocks antigen presentation by DCs, ultimately maintaining immune tolerance. DC-T cell crosstalk mainly involves chemokines, surface molecules (TonEBP, NFATc1), the PD-L1/PD-1 signalling axis, and the TGF-β signalling axis. In addition, DC-T cell crosstalk in RA is affected by glycolysis, reactive oxygen species, vitamin D, and other factors. These factors lead to the formation of an extremely complex regulatory network involving various mechanisms. This article reviews the key immune targets of DC-T cell crosstalk and elucidates the mechanism of DC-T cell crosstalk in RA to provide a basis for the treatment of patients with RA.

## Introduction

Rheumatoid arthritis (RA) is a chronic inflammatory disease characterised by joint pain and swelling, synovial hyperplasia, cartilage damage, and bone destruction. RA is closely related to an imbalance in immune cell subsets, immune dysfunction, and excessive activation of immune cells; however, its pathogenesis is complex and has not been fully elucidated [1]. Previous studies have shown that it is difficult for the body to maintain immune tolerance when peripheral regulatory T cells (Tregs) are deficient or dysfunctional. Genetic and environmental factors jointly induce abnormal activation of immune cells. Activated CD4^+^ T cells in the lymph nodes enter the peripheral blood and are transported to the joints under the action of chemokines, become aggressive effector cells, and destroy the immune balance of the body. This results in bone and cartilage damage in diseased joints. In addition, dendritic cells (DCs) and other immune cells enter the affected joints to participate in immune regulation. Activated DCs induce CD4^+^ T cells to differentiate into Th1, Th2, Th17, Tregs, follicular helper T (Tfh), and other cell subtypes that participate in the inflammatory cascade through various inflammatory cytokines. A large number of activated T cells inhibit the proliferation of DCs by secreting negative feedback cytokines, thereby maintaining the immune balance in the body [2]. Compared to other immune cells, the number of DCs is small, but DCs are distributed in all tissues and organs of the body and they play an important role in regulating human immune function. Tolerogenic dendritic cells (tolDCs) can induce Treg proliferation and exert protective effects. The induction of stable tolerance is an effective strategy for treating RA. The signal transduction involved in DC-T cell crosstalk mainly includes chemokines, surface molecules (TonEBP, NFATc1), the PD-L1/PD-1 signalling axis, and the TGF-β signalling axis [3–7]. Therefore, the mechanism of DC-T cell crosstalk is crucial for inducing RA and maintaining immune tolerance. Regulating DC-T cell crosstalk to delay the occurrence and development of RA is the main direction of future research. In addition, DC-T cell crosstalk in RA is affected by a variety of factors, such as glycolysis, reactive oxygen species (ROS), vitamin D, a hypoxic environment, and gut microbes [8–12], which create an extremely complex regulatory network between various mechanisms. Therefore, here, we comprehensively elucidated the mechanism of DC-T cell crosstalk in RA by analysing the signalling axes, chemokines, and surface molecules related to DC-T cell crosstalk. We discuss the factors affecting DC-T cell crosstalk in RA to provide a theoretical basis for future scientific research.

## Biological characteristics of DCs

DCs are powerful antigen-presenting cells (APCs) that perform multiple immune regulatory functions. The innate and adaptive immune responses mediated by DCs are of great significance in autoimmune diseases. Under physiological conditions, DCs can recognise, process, and present antigens to naïve T cells to stimulate their proliferation and differentiation. Additionally, DCs can initiate immune responses or maintain immune tolerance through cell–cell contact and the transmission of cytokine regulatory signals. However, the specific immunomodulatory function of DCs in the body depends on their phenotypic characteristics and maturation status [13]. In the stable phase, most DCs are immature and are present in peripheral tissues. Although immature DCs (imDCs) express low levels of costimulatory molecules and cytokines, which slow the activation of T cells and play an immunosuppressive role, they have strong migration and antigen phagocytosis abilities. When confronted with foreign antigens, such as viruses, bacteria, and damaged tissues, imDCs are regulated by proinflammatory factors, such as prostaglandin E2 (PGE2), tumour necrosis factor-ɑ (TNF-ɑ), and lipopolysaccharide (LPS). imDCs up-regulate the expression of costimulatory molecules, such as CD40 and CD80. CD86 and major histocompatibility complex (MHC) I/II expression gradually develop and the DCs mature through a series of complex physiological processes, such as specific signal transduction and transcriptional programming. Mature DCs (mDCs) can produce a large number of cytokines, such as interleukin-1β (IL-1β), IL-6, TNF-ɑ, chemokines (CCL3, CCL4, CCL5) and the homing receptor CCR7, which induce the migration of T cells from peripheral tissues to the T-lymphoid region of secondary lymphoid tissues. This promotes naïve T cell activation [14]. When pathogen-derived antigens migrate, mDCs may carry self-antigens, leading to non-inflammatory responses and immunosuppression. Therefore, mDCs can be classified as either immunogenic DCs or tolDCs. The expression levels of MHC and costimulatory molecules are lower on the surface of tolDCs than immunogenic DCs, resulting in a tolerant phenotype. Recently, tolDCs have been widely used in basic and clinical research on autoimmune diseases, organ transplantation, and tumours. Understanding the mechanisms of tolerance development and the optimal method for inducing stable tolerance may open up new therapeutic approaches for these diseases [15].

In recent years, the utilisation of high-throughput single-cell sequencing techniques has progressively unveiled further heterogeneity in the lineage characteristics and cellular states of dendritic cells (DCs). This implies that if a population of DCs exhibits distinct developmental pathways and transcription factors, it signifies the lineage characteristics of a subgroup within the DCs. Consequently, DCs can be categorised into various subtypes, including classical DC1 (cDC1), classical DC2 (cDC2), DC3, plasma-like DCs (pDCs), and monocyte-derived DCs (moDCs), based on their cellular and molecular ontogeny [16]. Previous studies have shown that the cDC1 population, mainly CD141^+^ DCs in humans and CD8α^+^ DCs and CD103^+^ DCs in mice, can initiate MHCi-CD8^+^ T cells to degrade endogenous antigens into antigen peptides and load them on MHCI molecules. Under the action of transcription factors, such as IRF8, Id2, and Batf3, cDC2s are presented to CD8^+^ T cells to participate in the immune response, while CDc2s initiate the MHCii-CD4^+^ T cell pathway, which takes up antigens and loads them on MHC II molecules through endocytosis and phagocytosis. MHC II molecules are present in CD4^+^ T cells under the action of IRF4 and participate in the immune response. In contrast to cDCs, pDCs in the blood migrate directly through high endothelial vessels into regions enriched with T lymphocytes in lymphoid organs. The main receptors driving cell migration are L-selectin (CD62L) and CCR7. pDCs that migrate to secondary lymphoid organs can secrete large amounts of type I interferon (IFN-I) and other inflammatory cytokines (such as TNF-ɑ and IL-6) to participate in the antigen cross-presentation process and play an antiviral immune function when stimulated by a viral infection or methylated nucleotides. Although the ability of pDCs to take up antigens remains unclear, mature pDCs can play an antigen presentation role by regulating their surface costimulants and the expression levels of MHC II molecules [17]. In contrast to the above DC-derivation pathway, peripheral blood monocytes can differentiate into moDCs in the presence of granulocyte–macrophage colony-stimulating factor (GM-CSF) and IL-4. However, moDCs do not have unique surface markers and largely overlap with macrophage surface markers. MoDCs do not functionally transport antigens to lymph nodes or activate T cells; therefore, it is not clear how moDCs induce nascent T cells to participate in the immune response [18].

## Biological characteristics of T cells

T cells differentiate into CD4^+^ and CD8^+^ T cells after gene rearrangement, T cell receptor (TCR) recognition, and selection. CD4^+^ T cells differentiate into Th1, Th2, Th22, Th17, Tregs, and Tfh cells when stimulated with different cytokines. The body coordinates various CD4^+^ T cell subsets to complete the immune response. During CD4^+^ T differentiation, IL-12 stimulation activates its own transcription factor, STAT4; promotes the activation of the Th1-cell-specific transcription factor, forkhead box P3 (FOXP3); and induces CD4^+^ T cells to differentiate into Th1 cells. Th1 cells are involved in immune regulation by secreting cytokines, such as IL-2, IFN-γ, TNF-α, and GM-CSF [19]. During the differentiation process, the Th2-cell-specific transcription factor, GATA-binding protein 3 (GATA3), can inhibit Th1 cell differentiation by antagonising T-bet. Similarly, Th2 cell differentiation is antagonised by the Th1 cell transcription factor, T-bet. The cytokines secreted by Th1 and Th2 cells promote the secretion of their own factors and also antagonise the production of cytokines. Th2 cells mainly secrete cytokines, such as IL-4, IL-10, and IL-13, which are involved in humoral immunity. As a positive feedback factor, IL-4 can also induce the phosphorylation of the transcription factor, STAT6, and further upregulate the expression of GATA3. IL-4/STAT6/GATA3 signalling continuously promotes the differentiation and expansion of Th2 cells [20]. Th22 cells are a subset of Th1, Th2, and Th17 cells. They mainly secrete the cytokines, IL-22 and TNF-α, and they express CCR4, Chemokine receptor 6 (CCR6), and CCR10, but do not secrete IFN-α, IL-4, IL-17, or other cytokines. IL-22 is important for the physiological function of Th22 cells, and its receptor is a transmembrane complex composed of IL-22R1 and IL-10R2. The activation of this receptor can activate downstream p38, JNK, ERK/MAPK, and Janus kinase/signal transducer and activator of transcription (JAK/STAT) signalling pathways to participate in the immune response [21, 22] Compared to other subsets, Th17 cells have a unique differentiation mechanism and they are regulated by a variety of cytokines. IL-6 plays an important role in the initial stages of the immune response. IL-6 and IL-6Rα recognise and bind to gp130 to form an active complex, which induces STAT3 phosphorylation. Furthermore, the up-regulation of the expression of the Th17 cell-specific transcription factor, retinoic acid-related orphan receptor (RORγt), ultimately promotes the differentiation of CD4^+^ T cells into Th17 cells. In addition, upregulation of the Treg marker molecules, SOCS3 and FOXP3, can prevent excessive differentiation of Th17 cells [23]. According to different cytokine environments, Th17 cells are divided into pathogenic Th17 cells and non-pathogenic Th17 cells. Pathogenic Th17 cells aggravate the inflammatory response by secreting the proinflammatory factors IL-17A, IL-17F, and IL-22, whereas non-pathogenic Th17 cells negatively regulate the inflammatory response by secreting the immunosuppressive factor IL-10. Th17 and Tregs inhibit each other during their generation and function. Tregs express the characteristic cell membrane marker, CD25 (IL-2Rα), and the nuclear transcription factor, FOXP3. They exert immunosuppressive functions mainly through metabolic disruption of target cells, the secretion of inhibitory cytokines, and direct cell–cell contact. CD25 competitively binds IL-2 to prevent the continuous proliferation of T cells, leading to the interruption of T cell differentiation [24]. Cytokines such as IL-10, IL-35, and TGF-β are secreted to negatively regulate the differentiation and function of T cells and exert immunosuppressive effects. Cytotoxic T lymphocyte-associated antigen 4 (CTLA4) is an inhibitory costimulatory receptor expressed by Tregs. It directly affects the immune response by competitively binding to the costimulatory molecules, CD80 and CD86, on the surface of APCs to reduce the number of effector T cells [25]. Tregs highly express lymphocyte activation gene 3 (LAG3, also known as CD223), which binds to the costimulatory molecule MHC II to limit the functional activity of APCs and effector T cells and induce immune tolerance. Tregs avoid damage caused by the excessive activation of T cells; therefore, they play a crucial role in maintaining immune homeostasis and self-tolerance [26]. Tfh cells are a subset of CD4^+^ T cells located in lymphoid follicles and peripheral immune tissues. They participate in the formation of germinal centres and assist B cell proliferation and differentiation, high-affinity antibody secretion, and immunoglobulin (Ig) class switching. Naive T cells activate the JAK/STAT signalling pathway in the presence of environmental IL-6 and IL-21, which promotes the expression of Bcl-6, a core transcription factor for Tfh cell differentiation. Bcl-6 promotes the expression of CXC family chemokine receptor 5 (CXCR5), inducible costimulatory molecule (ICOS), and programmed cell death receptor 1 (PD-1, also known as CD279) on the surface of Tfh cells, thereby inducing the differentiation of CD4^+^ T cells into CXCR5^+^ ICOS^+^ PD-1^+^ Tfh cells [27]. The immunomodulatory mechanisms of T cells have a profound effect on autoimmune diseases.

## Mechanisms of RA and DC-T cell crosstalk

In recent years, most studies have focused on the regulation of T cells by DCs, and the regulatory effect of T cells on DCs is worthy of further investigation. We summarised four aspects to explain the crosstalk mechanism between DCs and T cells: chemokines, surface molecules (TonEBP, NFATc1, MMP-9, GM-CSF), the PD-L1/PD-1 signalling axis, and the TGF-β signalling axis.

### Chemokines involved in DC-T cell crosstalk

**Fig. 1:**
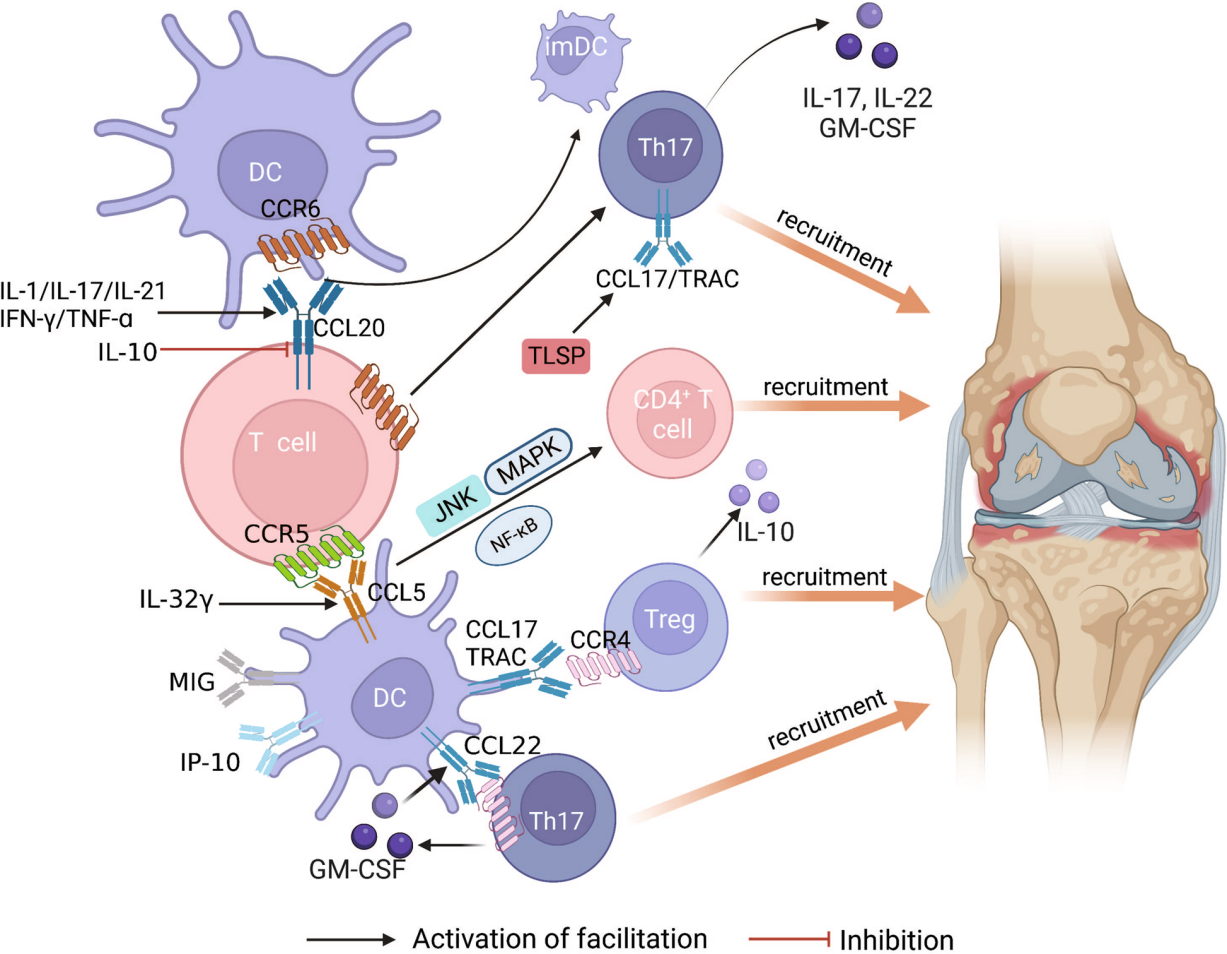
Chemokines involved in DC-T cell crosstalk. This picture summarises that chemokine ligand-receptors abundantly expressed in DCs and T cells play an important role in DC-T cell crosstalk in RA. Chemokines drive these immune cells to migrate to diseased joints and induce destructive immune responses. Black arrows indicate the promotion of proliferation and differentiation of CD4^+^T cell subtypes. Inhibition arrows in red indicate inhibition of chemokines

Leukocytes, inflammatory cells, and their products participate in T cell-mediated inflammation, which is the main cause of joint destruction and pain in patients with RA. Chemokines control leukocyte migration and aggregation. Chemokines are peptides that are approximately 8–14 kDa in size and are mainly divided into CCL, CXCL, and CX3CL. Their corresponding chemokine receptors can be divided into CXCR, CCR, CX3CR, and XCR [28]. An analysis of joint tissues from mice with collagen-induced arthritis (CIA) and peripheral blood from patients with RA revealed that a range of chemokines and their receptors are abundantly expressed in DCs and T cells, indicating that chemokine ligand receptors play an important role in DC-T cell crosstalk in RA. Compared to the control group, the expression level of CD209/CD14^+^ DC in the blood of patients with RA is elevated. This increase in CD209/CD14^+^ DC leads to the secretion of a significant amount of inflammatory factors, thereby exacerbating the inflammatory response in the joints. Furthermore, CD209/CD14^+^ DC expresses distinct chemokine receptors (CXCR3 and CCR7) and chemokine co-expression profiles (CXCR3/CXC5/CXCR5 and CCR6/CCR7/CXCR3/CXCR4), which facilitate the recruitment of CD209/CD14^+^ DC and immune cells to the affected joint [29]. Simultaneously, the study also ascertained the potential involvement of the JAK/STAT pathway in the recruitment of immune cells, while noting a reduction in chemokine receptor expression subsequent to the administration of JAK/STAT inhibitors in the course of treatment [29]. CCR6 is highly expressed in imDCs, Tregs, Th17 cells, and other immune cells in different tissue microenvironments, and CCR6 can drive the migration of these immune cells to CCL20-rich joints. The only known high-affinity homologue of CCR6 is CC chemokine ligand 20 (CCL20). Upregulation of CCL20 is associated with IL-1, IL-17, IL-21, IFN-γ, TNF-ɑ, and other cytokines, while IL-10 down-regulates the expression of CCL20 [3]. Staining localisation experiments have shown that CCL20 has the same chemotactic effect on imDCs, indicating that CCR6/CCL20 drives imDCs and Th17 cells to migrate to diseased joints and aggravates the inflammatory response [30]. Additionally, CCR6 expressed by CD4^+^ T cells inhibits the function of FOXP3 and promotes the differentiation of CD4^+^ T cells into Th17 cells, leading to an imbalance in the Th17/Tregs ratio and accelerating the development and progression of RA [31]. Another study found that IL-32γ induces the expression of various chemokines on DCs, including CCL2, CCL4, and CCL5, and that IL-32γ had the strongest regulatory effect on CCL5 in a dose-dependent manner. CCR1, CCR3, and CCR5, the receptors of CCL5, all exhibit transendothelial chemotaxis; however, CCR5 is more likely to regulate the diffusion of effector T cells into tissues. The chemotactic effect of CCL5 on T cells mainly depends on the JNK and nuclear factor κB (NF-κB) signalling pathways. This indicates that IL-32γ regulates the JNK and NF-κB signalling pathways by inducing CCL5 expression in DCs, which ultimately leads to the recruitment of T cells to the joint [32]. Previous studies have shown that Th1 cells in the synovial fluid of patients with RA highly express CXCR3 and its ligands and that CXCR3 binds to the ligands CXCL9/MIG and CXCL-10/IP-10 to exert chemotactic effects. In another study, there are many activated CD1c^+^ mDCs also secreted high levels of MIG, IP-10, and CCL17/TRAC. CCL17/TRAC is a selective ligand for CCR4 and is mainly expressed by Treg, Th2, and Th17 cells. CCR4 contributes to the migration of these CCL17/TRAC-rich T cells to inflamed tissues [33]. Li et al. [34] conducted in vitro studies wherein lipopolysaccharide (LPS) was employed to induce the differentiation and maturation of bone marrow-derived dendritic cells (BMDCs), followed by a 24-h intervention with apigenin (API). The findings revealed that API effectively suppressed the functional maturation of BMDCs and the chemotaxis of immune cells, potentially due to a reduction in the expression level of CCR4. The in vivo research findings demonstrated a reduction in the secretion of serum pro-inflammatory factors in CIA mice following API treatment, as well as a decrease in the expression of co-stimulatory molecules and MHC class II molecules on DCs. Although DCs maintained their expression of CCR5 and CCR7, the expression level of CCR4 decreased. Furthermore, API treatment hindered the recruitment of immune cells in joints, indicating a potential correlation between the alleviation of joint symptoms in CIA mice and the API-mediated inhibition of CCR4. Hillen et al. [35] used thymic stromal lymphopoietin (TLSP) to activate cD1c cDCs in the synovial fluid to produce the chemokine TRAC in vitro. Compared with untreated DCs, TLSP-DCs produced more TNF-ɑ. When chemotaxis was blocked, the expression of the inflammatory factors IFN-γ and IL-17 was reduced. It has been suggested that the immune responses of Th1 and Th17 cells are regulated by TRAC. As the expression of IL-4 was not detected in this experiment, whether Th2 cells, which widely express CCR4, migrate to the TRAC gradient requires further study. Th17 cells in mice with CIA express CCR4 and secrete IL-17A and GM-CSF, which promote CCL22 expression in DCs. CCL22 interacts with CCR4 to drive Th17 cell recruitment. This further verifies that the differentiation and proliferation of Th17 cells are inhibited in CCR4-deficient mice with CIA. The CCR4/CCL22 axis plays a key role in the recruitment and expansion of Th17 cells in mice with CIA. Reports indicate that CCR9 and its ligand CCL25 have been observed in the synovium of individuals with RA, with CCR9 expression specifically identified in DCs. The administration of CCR9 antagonists or the suppression of CCR9 expression has demonstrated the potential to alleviate arthritis symptoms in mice. However, the precise mechanism by which CCR9 and its ligand modulate the activity of DCs and T cells in the context of RA necessitates further investigation [36]. Li et al. [37] used the natural polyphenolic compound naringenin (5,7,4-trihydroxyflavone) to treat mice with CIA and found that it effectively improved joint disease severity and inhibited the production of costimulatory molecules, proinflammatory cytokines (IL-6I, L-12, and TNF-ɑ), chemokines (MCP-1 and MIP-1ɑ), and the priming of specific T cells. Naringenin not only blocks the maturation of DCs induced by LPS, but also selectively inhibits the proliferation of a subset of T cells and the production of inflammatory factors. In addition, the inhibition of NF-κB and MAPK signalling pathways, which are involved in regulating the differentiation and maturation of DCs, may be closely related to the mechanism of action of naringenin. In summary, it can be concluded that both DC and T cells exhibit a significant expression of numerous chemokines and engage in interactions with their respective receptors, thereby facilitating the migration of immune cells towards afflicted joints. This process ultimately results in immune dysfunction and the initiation of detrimental immune responses (Fig. 1). Chemokines and their receptors may be used as entry points to regulate DC-T cell crosstalk in RA and to identify new target molecules that regulate CCR4, CCR5, CCR6, and their ligands, which can be used as the basis for screening RA-protective drugs and providing new strategies for the treatment of RA.

### Surface molecules involved in DC-T cell crosstalk

**Fig. 2:**
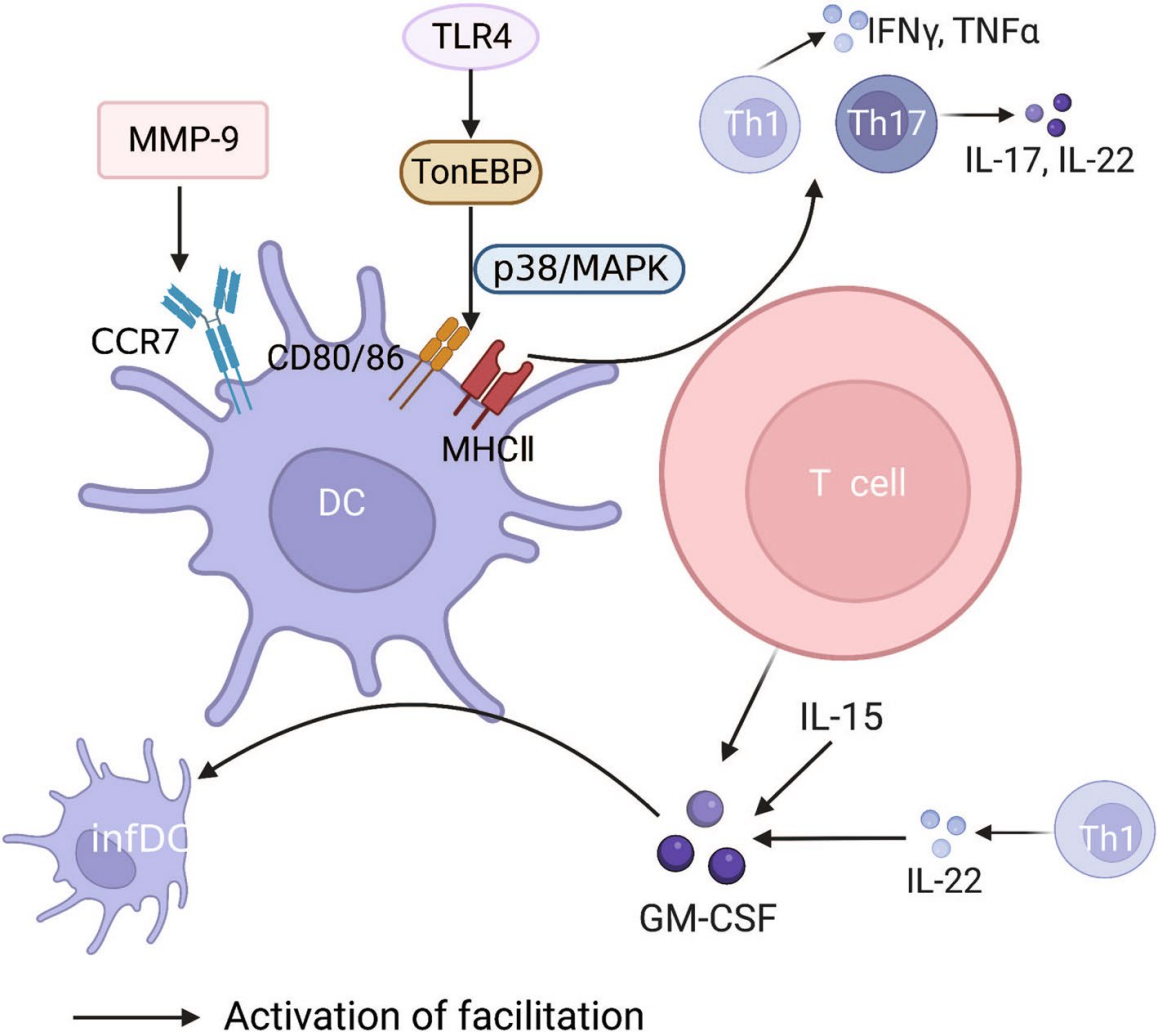
Surface molecules involved in DC-T cell crosstalk. This picture summarises the surface molecules involved in DC-T cell crosstalk in RA, with black arrows indicating that they promote the proliferation and differentiation of CD4^+^T cell subtypes

Nuclear-factor-activated T (NFAT) cells are involved in inflammation and autoimmune diseases. TonEBP (NFAT5) is a central regulator that promotes the differentiation and maturation of DCs. TonEBP upregulates the expression of costimulatory molecules and MHC II by activating the p38/MAPK signalling pathway, which subsequently promotes the differentiation of CD4^+^ T cells into Th1 and Th17 cells. When TonEBP is deficient, the differentiation and maturation of DCs and CD4^+^ T cells are inhibited and foot swelling symptoms are relieved in mice with CIA [4]. In addition, NFAT cells are also involved in the process of RAW264.7 cells differentiating into osteoclasts induced by receptor activator of nuclear factor-κb ligand (RANKL) in RA. Zeng et al. [5] used aconitine to treat RAW264.7 cells and found that different doses of aconitine had no significant effect on the activity of these cells. However, aconitine dose-dependently inhibited RANKL/NF-κB and RANKL/NFATc1 signalling pathways in RAW264.7 cells and reduced the expression levels of osteoclast-specific genes (MMP-9 and CtsK) and DC-specific transmembrane protein (DC-STAMP). MMP-9 is a major factor in the destruction of the mineral and collagen matrix in joints and is also a key molecule in promoting the migration of DCs. The treatment of mice with CIA with an MMP-9 inhibitor significantly inhibited the development of RA. Thus, MMP-9 inhibition may be an effective approach for RA treatment [38]. The main source of GM-CSF in RA is CD4^+^ T cells, which can promote the differentiation of infDCs and exhibit a strong allogeneic proliferative capacity. The upregulation of GM-CSF expression correlates with IL-15 and IL-12 secretion by Th1 cells. These cells promote disease through a positive feedback loop in a chronic inflammatory state [39]. As mentioned above, various signalling pathways centred on the PD-L1/PD-1 signalling axis, TGF-β1, and chemokines and their ligands explain most of the mechanisms of DC-T cell crosstalk in RA. Surface molecules, such as TonEBP, NFATc1, MMP-9, and GM-CSF, are also involved in DC-T cell crosstalk. However, only a few studies have verified the unidirectional regulation of DCs by T cells via these signalling molecules (Fig. 2).

### DC-T cell crosstalk and the PD-L1/PD-1 signalling axis

The PD-L1/PD-1 signalling axis plays an important regulatory role in the induction of peripheral immune tolerance. PD-1 is a type I transmembrane protein with a size of approximately 50–55 kDa. It is composed of an extracellular domain, a transmembrane domain, and a cytoplasmic domain. It belongs to the CD28 family of receptors and is widely expressed on the surfaces of DCs and T cells. PD-1 interacts with its ligand, PD-L1, to inhibit the expression of TCR signal transduction and costimulatory molecules. It has a negative regulatory effect on the immune response and can prevent autoimmune diseases caused by persistent inflammatory reactions [40]. Two tyrosine residues, which are located in the immunoreceptor tyrosine inhibitory motif (ITIM) and the immunoreceptor tyrosine switching motif (ITSM) in the cytoplasmic domain, are essential for PD-1 to suppress host immune function. Upon antigen stimulation, the tyrosine residues in the ITIM and ITSM are phosphorylated, which further activates protein tyrosine phosphatase 2 (SHP2) [41]. Downstream spleen tyrosine kinase (Syk) and phosphatidylinositol-3 kinase (PI3K) on the surface of T cells are dephosphorylated and activated, blocking TCR function, and affecting the transcription and translation of cytokines required for the normal differentiation and proliferation of T cells [42]. Generally, PD-1^+^ DCs preferentially upregulate the expression of the Treg nuclear transcription factor FOXP3, promote the differentiation of naive T cells into Tregs, and inhibit the differentiation of other CD4^+^ T cells. Tregs limit the functional activity of effector T cells and inhibit immune responses in various ways. Notably, the PD-L1/PD-1 signalling axis is also involved in the process of T cells negatively regulating the differentiation and maturation of DCs, inhibiting the secretion of proinflammatory factors, and inducing the immune tolerance of PD-1^+^ DCs. Therefore, the PD-L1/PD-1 signalling axis is a bidirectional communication mechanism for DC-T cell crosstalk. In addition, the PD-L1/PD-1 signalling axis is also regulated by MAPK, hypoxia-inducible factor (HIF), NF-κB, STAT3/5, and other signalling pathways [43].

**Fig. 3:**
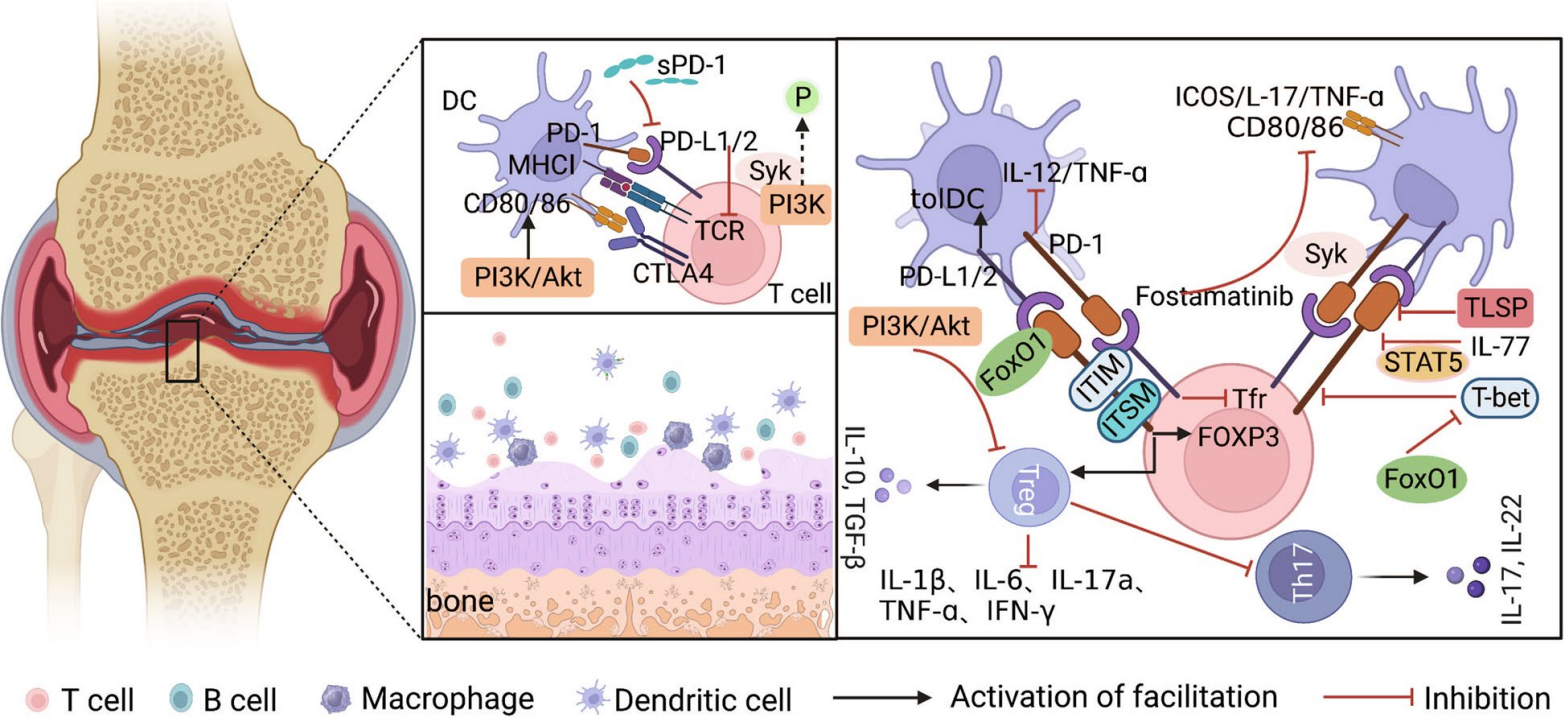
DC-T cell crosstalk and the PD-L1/PD-1 signalling axis. This figure summarises the PD-L1/PD-1 signalling axis to maintain immune balance by regulating the function of DCs and T cells. Patients with RA exhibit heightened infiltration of immune cells within their joints, accompanied by immune system dysregulation and impaired T cell function. The activation of synovial fibroblasts is directly facilitated by Th17 through the secretion of IL-17 and IL-22, resulting in an exaggerated immune response in RA. Furthermore, Th17 indirectly exacerbates synovitis and bone destruction in RA by promoting the generation of osteoclasts and subsequent bone resorption. However, there exists a functional inhibition between Th17 cells and Treg, and any abnormalities in the quantity or function of Treg can trigger an immune cascade reaction, resulting in the production of inflammatory factors such as IL-1β, IL-6, and TNF-α, which can lead to joint cartilage damage. PD-1 interacts with its ligand PD-L1 to promote the differentiation of naive T cells into Tregs, which in turn inhibit the differentiation of other CD4^+^T subsets. It has a negative regulatory effect on immune response and can avoid spontaneous immune diseases caused by a persistent inflammatory response. Black arrows indicate the promotion of proliferation and differentiation of CD4^+^T cell subtypes. Inhibition arrows in red indicate inhibition of proliferation and differentiation of CD4^+^T cell subtypes

Previous studies have found that the expression levels of PD-L1 and PD-1 are significantly decreased in mouse models of collagen-induced arthritis, which in turn, increases the secretion of proinflammatory factors, causes destructive immune responses, and aggravates damage to diseased joints [6]. Soluble PD-1 (sPD-1) can inhibit the function of the PD-L1/PD-1 signalling axis in RA patients to induce immune tolerance, leading to excessive activation of the T cell immune response and driving the occurrence and development of RA [44]. Syk is a core protein of the PD-L1/PD-1 signalling axis. Fostamatinib, a Syk inhibitor, was used by Platt to improve inflammation in patients with RA [45]. The results showed that fostamatinib did not inhibit CD4^+^ T cell proliferation in mice with CIA. In vitro experiments showed that fostamatinib reduced the duration and number of initial DC-T cell interactions; inhibited the proliferation and differentiation of CD4^+^ T cells; and reduced the expression levels of PD-1, ICOS, costimulatory molecules, IL-17, and TNF-ɑ. This analysis revealed that the reason for the discrepancy between in vivo and in vitro experimental results may be that the active metabolite of fostamatinib, R406, did not reach the target cells at the right time or at an appropriate concentration. Previous studies have shown that TSLP promotes the expression of PD-L1 mRNA in synovial fluid and peripheral blood of patients with RA and inhibits the secretion of antigen-presenting molecules (HLA II and CD1c), costimulating factors (CD40, CD80, and CD86), and T cell chemokines. Blocking the interaction between PD-1 and PD-L1 reverses T cell hyporesponsiveness to TSLP-mDCs. In addition, IL-7 down-regulates the expression of PD-1. IL-7 may reverse the inhibitory effect of the PD-L1/PD-1 signalling axis on T cell physiological function by activating STAT5, which is the key mechanism by which IL-7 regulates T cell immune responses; however, the involvement of other immune mechanisms induced by IL-7 cannot be excluded [46]. Samarpita et al. [47] found that emodin downregulates the expression of the costimulatory molecules CD86 and MHC II by inhibiting the PI3K/Akt signalling pathway, which further promotes the binding of FoxO1 to the PD-1 promoter and enhances the anti-inflammatory effect of Tregs/IL-10. FoxO1 is a key regulator of the PD-L1/PD-1 signalling axis. FoxO1 inhibits the expression of the Th1-cell-specific transcription factor, T-bet. However, T-bet is an inhibitor of the PD-L1/PD-1 signalling axis; therefore, FoxO1 can indirectly promote the accumulation of PD-1 in T cells, indicating that regulation of the PD-L1/PD-1 signalling axis by the PI3K/AKT/FoxO1 signalling pathway is crucial for maintaining the Th17/Treg cell balance. Hu et al. [48] transferred the PD-L1 gene into bone marrow mesenchymal stem cells and induced continuously high expression levels of PD-L1 protein in mice with CIA, resulting in a decrease in the number of DCs, Th1 cells, and Th7 cells and an increase in the number of Tregs. Inhibition of the secretion of proinflammatory factors, such as IL-1β, IL-6, IL-17a, TNF-β, and IFN-β, and promotion of the secretion of the anti-inflammatory factor, IL-10, contribute to the restoration of immune tolerance and ultimately, effectively reverse articular cartilage damage in mice with CIA. In summary, patients with RA exhibit heightened infiltration of immune cells within their joints, accompanied by immune system dysregulation and impaired T cell function. The activation of synovial fibroblasts is directly facilitated by Th17 through the secretion of IL-17 and IL-22, resulting in an exaggerated immune response in RA. Furthermore, Th17 indirectly exacerbates synovitis and bone destruction in RA by promoting the generation of osteoclasts and subsequent bone resorption. However, there exists a functional inhibition between Th17 cells and Treg, and any abnormalities in the quantity or function of Treg can trigger an immune cascade reaction, resulting in the production of inflammatory factors such as IL-1β, IL-6, and TNF-α, which can lead to joint cartilage damage (Fig. 3). The interaction between PD-1 and its ligand PD-L1 can facilitate the differentiation of immature T cells into Treg, thereby suppressing the differentiation of other CD4^+^ T subsets. This negative regulatory effect on the immune response prevents the occurrence of spontaneous immune diseases caused by prolonged inflammatory responses (Fig. 3). Therefore, the PD-L1/PD-1 signalling axis is important for maintaining immune balance, and identifying effective therapeutic drugs targeting the PD-L1/PD-1 signalling axis may be the main research field for the clinical treatment of RA in the future. The identification of more biological inhibitors (such as small molecules used in traditional Chinese medicines or effective extracts of traditional Chinese medicines) will be the focus of future research.

### DC-T cell crosstalk and the TGF-β signalling axis

TGF-β is a multi-effector molecule that regulates immune responses. The most immunologically relevant isoform is TGF-β1. Previous studies have found that TGF-β plays a key regulatory role in the differentiation and function of DCs, but its role in regulating T cells in different disease models is controversial. Multiple injections of TGF-β into the joints of healthy mice can induce an inflammatory response, leading to the development of osteophytes and synovial hyperplasia in the joints [49], while in another study, TGF-β showed the opposite effect. Park et al. [7]. used bone marrow mesenchymal stem cells transduced with an adenovirus vector directing the expression of TGF-β to treat CIA. The data showed that the expression levels of proinflammatory factors, such as IL-6, IL-17, and TNF-ɑ, were down-regulated, and the expression of the anti-inflammatory factor, IL-10, and the Treg-specific transcription factor, FOXP3, was promoted, resulting in a decrease in the Th17/Treg cell ratio in the peritoneum and spleen. Tregs effectively impede the proliferation of pathogenic effector T cells, ultimately reducing cartilage destruction and bone erosion and delaying RA progression. It has been reported that the process of the induction of FOXP3 expression by TGF-β to promote Treg differentiation can be inhibited by IL-27. This effect may be achieved via STAT3, and it is independent of the inhibitory effect of IL-27 on IL-2, which is required for Treg differentiation [50]. It has been reported that TGF-β promotes the differentiation of CD4^+^ T cells into Th9 cells, Th17 cells, Tregs, and Tfh cells, and it can also block the production of Th1 and Th2 cells. Therefore, TGF-β is both an inhibitor and a promoter of inflammation, with opposite effects on the regulation of the inflammatory response. It was later found that the immunomodulatory function of TGF-β on CD4^+^ T cell differentiation may depend on the cytokine microenvironment and the stage of T cell differentiation, but the specific mechanisms remain unclear [51].

**Fig. 4:**
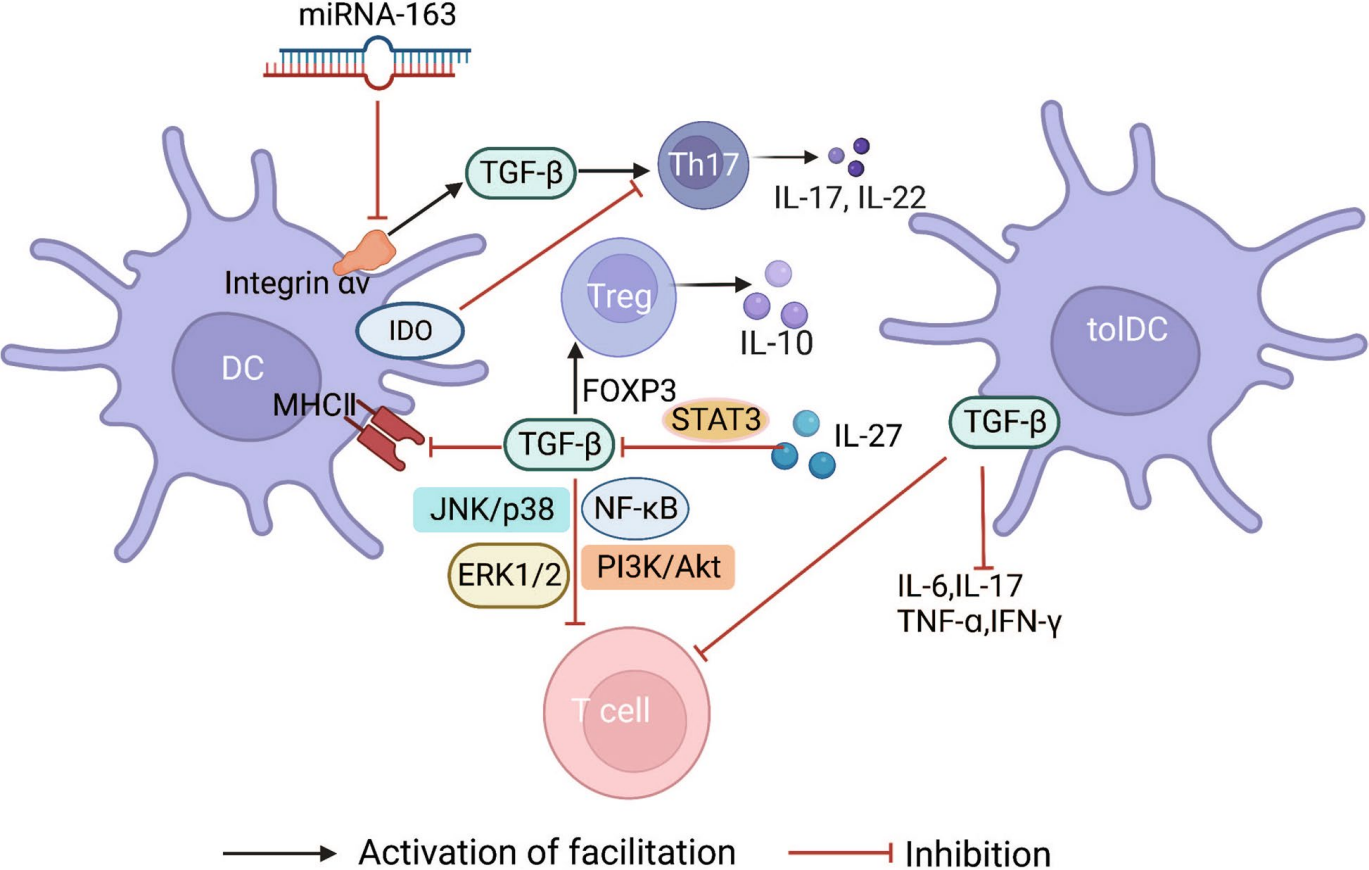
DC-T cell crosstalk and the TGF-β signalling axis. This picture summarises that the TGF-β signalling axis plays an important regulatory role in DC-T cell crosstalk in RA and that TGF-β has two completely opposite effects on the regulation of inflammatory responses that may depend on the cytokine microenvironment and the stage of T cell differentiation. Black arrows indicate the promotion of proliferation and differentiation of CD4^+^T cell subtypes. Inhibition arrows in red indicate inhibition of proliferation and differentiation of CD4^+^T cell subtypes

CD11c^+^ stimulates the differentiation and proliferation of Th17 cells in the early immune response in RA patients, and the expansion of Th17 cells requires DCs to express integrin αv and RORγt. Integrin αv can excessively activate downstream TGF-β, leading to Th17/Treg cell imbalance. It has also been observed that the CD11c^+^αv^+^ DCs/CD11c^+^ DCs ratio in patients with RA is positively correlated with the ratio of Th17/Treg cells, indicating that CD11c^+^αv^+^ DCs are more conducive to the differentiation of CD4^+^ T cells into Th17 cells. In addition, the expression level of *miR-363* is down-regulated in RA DCs. *miR-363* participates in immune responses by negatively regulating ITGAV, which encodes integrin αv. Therefore, the *miR-363*/integrin αv/TGF-β signalling pathway plays an important role in DC-T cell crosstalk, suggesting that promoting *miR-363* expression may be a potential mechanism to suppress destructive immunity [52]. Previous studies have reported that TGF-β induces the expression of the tryptophan catabolase indoleamine 2, 3-dioxygenase (IDO) on DCs through the non-canonical NF-κB pathway, which has similar effects to integrin αv, which IDO is mainly expressed on DCs, and its upregulation promotes the immune tolerance of DCs and inhibits the differentiation and proliferation of T cells [53, 54]. In recent years, tolDCs produced by dexamethasone and vitamin D3 modulation have been found to have potent inhibitory effects on CD4^+^ T cells and are crucial for the treatment of RA and autoimmune diseases. tolDCs express higher levels of TGF-β1 than mDCs. Although TGF-β1 is not the main factor that regulates the immune response of CD4^+^ T cells, these cells are still regulated by TGF-β1 in tolDCs. Data show that TGF-β1 has a strong inhibitory effect on TNF-ɑ, IFN-γ, and other proinflammatory factors, and CD4^+^ T cell proliferation is affected by the costimulatory molecule, CD28. In the absence of CD28, TGF-β1 inhibits naïve T cell proliferation, but enhances T cell proliferation and differentiation in the presence of CD28; therefore, the regulation of CD4^+^ T cells by tolDCs via TGF-β1 may be a key route to restore immune tolerance in RA [55]. Oh et al. [56] used a TGF-β agonist (T74) to treat mice with CIA. Compared to the control group, mice with CIA injected with T74 showed no inflammatory cell infiltration or synovial lesions, and the joint space was normal, without bone or cartilage damage. In addition, the expression levels of proinflammatory cytokines, costimulatory molecules, MHC class II molecules and MDC-specific markers (pdlim4 and Rsad2) in T74-DCs were significantly reduced, and Th1/Th17 differentiation was also restricted, suggesting that T74 inhibited MDC formation and maintained immune tolerance through competitive binding to TGF-βRI. However, T74 did not act on the downstream Smad pathway of TGF-β, but on the JNK/p38, ERK1/2, NF-β B, and PI3K/Akt signalling pathways in DCs. Compared with the classical TGF-β/Smad signalling pathway, these pathways have a stronger effect on inhibiting the differentiation and maturation of DCs and T cells. In conclusion, the TGF-β signalling pathway axis exhibits divergent effects on the regulation of the inflammatory response in RA, potentially contingent upon the specific cytokine microenvironment and stage of T cell differentiation (Fig. 4). Future studies may include regulation of the expression of TGF-β protein or the downstream signalling pathways of TGF-β, such as JNK/p38, ERK1/2, NF-κB, TGF-β/Smad, and PI3K/Akt, to maintain the immune tolerance of DCs and T cells and prevent excessive immune responses. This may be a key basis for the future development of drugs to treat RA.

## Factors influencing DC-T cell crosstalk in RA

DC-T cell crosstalk in RA is affected by a variety of factors, such as glycolysis, ROS, vitamin D, a hypoxic environment, and gut microbes, which form an extremely complex regulatory network between the various mechanisms [8–12].

### Glycolytic pathway

The energy required to maintain the physiological functions of cells is primarily derived from glucose metabolism. Glucose undergoes glycolysis in the cytoplasm and the resulting pyruvate enters the tricarboxylic acid cycle for oxidative phosphorylation to produce more ATP. During inflammation, glycolysis is the main pathway of glucose metabolism in immune cells, and oxidative phosphorylation is restored when inflammation subsides. Although the efficiency of glycolysis for ATP production is low, glycolytic intermediates are important factors that activate immune cells [8]. Reprogramming glucose metabolism from oxidative metabolism to glycolysis is required for DC activation, phenotype maintenance, and migration. TLR signalling rapidly activates DCs and promotes the phosphorylation of TANK-binding kinase 1 (TBK1)/nuclear factor-κb kinase inhibitor (IKKε)/Akt, which subsequently upregulates the expression of HK2 and LDHA via p-Akt/mTORC1 and HIF-1ɑ [57]. HK2 enrichment in mitochondrial ion channels contributes to T cell activation by DCs [58]. In addition, defective glycolysis in T cells in RA leads to the accumulation of NADPH and ROS from glucose via the pentose phosphate pathway. These excess reducing products can inhibit the activation of REDOX kinases associated with T cell activation, allowing T cells to bypass the G2/M cell cycle checkpoint, ultimately leading to T cell hyperproliferation [59]. The differentiation of CD4^+^ T cell subsets depends on different metabolic pathways. Th17 cells undergo glycolysis for proliferation and differentiation, whereas Treg differentiation depends on oxidative phosphorylation and fatty acid oxidation. The treatment of MoDCs with explant-conditioned medium (ECM) derived from RA synovial tissue biopsy cultures restores the synovial microenvironment. The results showed increased expression levels of proinflammatory cytokines, chemokines, and costimulatory molecules, accompanied by alterations in DC maturation and glucose metabolism. ECM treatment changes cell bioenergy metabolism in MoDCs, mainly by increasing the levels of the amino acid transporter, CD98, and changing the glycolytic metabolic profile. The pharmacological inhibition of STAT3 inhibits the expression of glycolytic genes in ECM-treated MoDCs, indicating that this change may be related to the involvement of STAT3 in regulating DC maturation and metabolic reprogramming. Excess IL-6 in ECM has been hypothesised to be an upstream target of STAT3, but this has not been experimentally verified [60]. Hexokinase (HK) is the first key enzyme involved in glycolysis and it has four isoforms. The HK2 isoform is highly expressed in Th17 cells and is a key rate-limiting factor for the proliferation and differentiation of Th17 cells. To study the regulatory effects of 3-bromopyruvate (BrPA), a specific inhibitor of HK2, on RA immune cells, Okano et al. [61] treated SKG mice (an RA model) with BrPA. The data showed that BrPA promoted the proliferation of Tregs and inhibited the proliferation of Th17 cells and DCs, indicating a dual effect on immune cells. The inhibitory effect of BrPA on DCs is closely related to the stimulatory effect of TLR signalling, which promotes the association of mitochondria with HK2; however, the specific mechanism is not clear. It is speculated that the inhibition of glycolysis by BrPA increases the expression level of FOXP3, thereby inhibiting the expression of the Th17 cell-specific transcription factor, RORγt, and promoting the differentiation and proliferation of Tregs. In addition, it has been confirmed that IL-6 induces the overexpression of HIF-1ɑ and key glycolytic enzymes through the activation of mTOR during Th17 cell differentiation. This increases the efficiency of glycolysis [62]. The research [63] has indicated that within inflamed joints affected by RA, a state of severe oxygen deprivation, known as hypoxia, occurs. This hypoxic environment leads to the activation of cross-linked antibodies, which in turn trigger receptor 1 (TREM-1) on the surface of myeloid cells found on CD141^+^ DCs. This activation results in the rearrangement of the cytoskeleton, a process that proves advantageous for the formation of immune synapses and subsequent activation of T cells. The regulatory impact of TREM-1 on CD141^+^ DCs is contingent upon the presence of hypoxia, and fully developed synovial CD141^+^ DCs activate T cells by means of the interaction between p38 and TREM-1, ultimately leading to the production of IFN- γ. Furthermore, the interplay between synovial CD141^+^ DC and T cells has the potential to elicit additional stimulation of synovial fibroblasts, thereby instigating adhesion and invasive pathogenic mechanisms. Bian et al. [64] reported that the pyruvate dehydrogenase inhibitor, dichloroacetic acid (DCA), also ameliorates joint damage in mice with CIA by inhibiting glycolysis; however, they did not investigate whether DCA had an effect on the regulation of DCs or the Th17/Treg axis. Therefore, key enzymes in the glycolytic pathway may be effective targets for the treatment of autoimmune diseases and may become a key basis for the regulation of DC-T cell crosstalk by RA drugs in the future.

### ROS

Previous studies have shown that ROS produced by the body can promote the differentiation, maturation, and activation of DCs, and many oxidative autoantigens have been found in patients with autoimmune diseases, indicating that ROS can affect the function of T cells by changing the antigen structure. ROS production mainly depends on NADPH oxidase 2 (NOX2) [65]. Inhibition of NOX2 function results in decreased expression levels of cellular inflammatory cytokines and costimulatory molecules in DCs. Upon cytosolic phosphatidylation, Ncf1 interacts with Ncf2 and Ncf4 on canonical organelle phospholipids to attach to the membrane and then binds with NOX2 to form a complex that assists in electron transfer from NADPH to oxygen. As a result, large amounts of ROS and H_2_O_2_ are produced, which affect antigen synthesis and the MHC II molecule loading process in DCs. Antigen presentation is a key step in promoting T cell activation. Thus, ROS play an important role in innate immunity [9]. Similarly, NOX5 can regulate through the JAK/STAT pathway in moDC differentiation of proliferation [66]. Xiao et al. [67] used the natural product, *Piper longum* (PLM) to treat mice with CIA and found that PLM inhibited the maturation of bone marrow-derived DCs. The expression levels of costimulatory molecules (MHC-II and CD80/86), inflammatory factors (IL-6, IL-12, IL-2, TNF-ɑ, and IFN-γ), and chemokines (MCP-1) were decreased, accompanied by a reduction in T cell proliferation. In addition, PLM inhibited the maturation of DCs by reducing ROS levels. It also inhibited the ROS-mediated p38, NF-kB, JNK, and PI3K/Akt signalling pathways in DCs and affected the proliferation and differentiation of T cells, suggesting the importance of inhibiting ROS production in RA. It is well known that tolerogenic dendritic cell-derived exosomes (TolDex) are emerging modulators of autoimmune diseases [68]. Lee et al. [69] endowed TolDex with responsiveness to ROS production from physiological stimuli in RA by embedding the ROS-sensitive functional groups, thionone (TK) and polyethylene glycol (PEG), on its surface. The presence of PEG solves the problem of poor stability and low targeting of exosomes and helps maintain the integrity of the TolDex structure. The data showed that TolDex downregulated the expression levels of CD40, TNF-β, and IL-6; upregulated the expression level of TGF-β; and promoted Treg differentiation, suggesting that TolDex induces immune tolerance in RA. Because of the ROS sensitivity of TKDex, TKDex and TolDex have similar effects on immune regulation. In general, TolDex, which is reactive to ROS, is a potential agent for treating RA. Therefore, ROS are expected to become new targets for RA treatment in the future, and may also assist TKDex in regulating the immune response and be widely used in clinical practice.

### Vitamin D

Owing to the pleiotropic nature of vitamin D, its deficiency not only causes osteoporosis but also affects autoimmune diseases. The active form of vitamin D with immunomodulatory effects is 1,25-dihydroxyvitamin D3 (1,25-(OH)2D3), and it has been reported that the receptor of 1,25-(OH)2D3, VDR, is expressed on DCs and T cells. 1,25-(OH)2D3 reduces the expression levels of costimulatory molecules (CD40, CD80, and CD86), MHC class II molecules, and inflammatory cytokines (IL-12 and IL-23) in DCs. In turn, this inhibits the proliferation and differentiation of CD4^+^ T cells [10]. Specifically, 1,25-(OH)2D3 inhibits Th1 and Th17 cells and reduces the levels of key regulators of Th1 and Th17 cell differentiation, including IL-2, TNF-ɑ, IFN-γ, and RORγt, while 1,25-(OH)2D3 promotes Treg differentiation and proliferation. It can prevent an excessive immune response by inducing the specific transcription factor, FOXP3, and the anti-inflammatory cytokine, IL-10, to maintain the body's immune balance. In mouse models of RA, the induction of tolDCs is a key measure to delay disease progression, as it maintains the Th17/Treg cell balance. 1,25-(OH)2D3 induces tolDCs, suggesting that it is a potential agent for the treatment of RA [70]. In addition, air pollution is a risk factor for RA, and the harmful gas ozone absorbs ultraviolet (UVB) rays in the air, reducing the efficiency of sunlight to synthesise vitamin D in the skin and thereby, increasing the incidence of RA [71]. Clinical studies have shown that vitamin D supplementation can be used for the prevention and treatment of autoimmune diseases; however, its effectiveness remains unclear. Further observations of the therapeutic effects of vitamin D and the establishment of a reasonable strategy for vitamin D supplementation are needed.

## Conclusions and perspectives

RA is a chronic inflammatory disease and its main pathogenesis involves an imbalance in immune cell subsets and immune dysfunction. Th2 cells and Tregs differentiated from CD4^+^ T cells have immunosuppressive functions and interact with other CD4^+^ T cell subsets to maintain immune tolerance and prevent destructive inflammatory responses. When the regulatory role of peripheral Tregs is inhibited, many abnormally activated CD4^+^ T cells enter the peripheral blood and are transported to the joints under the action of chemokines. The inflammatory cascade results in bone and cartilage damage in the affected joints. The differentiation and proliferation of these CD4^+^ T cell subsets are regulated by DC surface molecules and antigen presentation, thus forming an intricate DC-T cell crosstalk mechanism that brings great challenges to clinical treatment and prognosis. DC-T cell crosstalk involves a variety of signalling pathways, chemokines, and surface molecules. The PD-L1/PD-1 signalling axis, TGF-β signalling axis, chemokines, and their ligands are the main mechanisms that destroy immune tolerance in RA. In addition, DC-T cell crosstalk is affected by various factors, such as glycolysis, ROS, vitamin D, a hypoxic environment, and intestinal microbes, and these factors may become hot topics for future research on the pathogenesis of RA. Drugs that regulate DC-T cell crosstalk and methods that induce stable tolDC production are effective ways to treat RA; however, these immune cells and their secreted inflammatory cytokines have different onset times. Future studies aimed at selecting a reasonable time node to target and regulate DC-T cell crosstalk without affecting other immune cells are warranted.

## Data Availability

Not applicable.

## Abbreviations

APCs: Antigen-presenting cells
CDCs: Classical DCs
CCR6: Chemokine receptor 6
CTLA4: Cytotoxic T lymphocyte-associated antigen 4
CXCR5: CXC family chemokine receptor 5
CIA: Collagen-induced arthritis
CCL20: CC chemokine ligand 20
DC: Dendritic cell
DC-STAMP: DC-specific transmembrane protein
DCA: Dichloroacetic acid
ECM: Explant-conditioned medium
Tfh: Follicular helper T
FOXP3: Forkhead box P3
GM-CSF: Granulocyte-macrophage colony-stimulating factor
GATA3: GATA-binding protein 3
HIF: Hypoxia-inducible factor
HK: Hexokinase
ImDCs: Immature DCs
IL-1β: Interleukin-1β
ITIM: Immunoreceptor tyrosine inhibitory motif
ITSM: Immunoreceptor tyrosine switching motif
ICOS: Inducible costimulatory molecule
LCs: Langerhans cells
LAG3: Lymphocyte activation gene 3
MHC: Major histocompatibility complex
MoDCs: Monocyte-derived DCs
NF-κB: Nuclear factor κB
NFAT: Nuclear-factor-activated T
IKKε: Nuclear factor-κb kinase inhibitor
NOX2: NADPH oxidase 2
PGE2: Prostaglandin E2
PDCs: Plasmacytoid DCs
PD-1: Programmed cell death receptor 1
PI3K: Phosphatidylinositol-3 kinase
PEG: Polyethylene glycol
RA: Rheumatoid arthritis
ROS: Reactive oxygen species
RORγt: Retinoic acid-related orphan receptor
RANKL: Receptor activator of nuclear factor-κb ligand
SHP2: Tyrosine phosphatase 2
Syk: Spleen tyrosine kinase
sPD-1: Soluble PD-1
Tregs: Regulatory T cells
TolDCs: Tolerogenic dendritic cells
TNF-ɑ: Tumour necrosis factor-ɑ
TCR: T cell receptor
TBK1: TANK-binding kinase 1
TolDex: Tolerogenic dendritic cell-derived exosomes
TK: Thionone
TLSP: Thymic stromal lymphopoietin
UVB: Ultraviolet
1,25-(OH)2D3: 1,25-Dihydroxyvitamin D3
